# Navigating global health diplomacy: challenges and opportunities in building a community of practice

**DOI:** 10.1186/s12992-025-01100-z

**Published:** 2025-03-12

**Authors:** Paul Rosenbaum, Carita Rehn, Karl Wennberg, Anders Nordström, Tobias Alfvén

**Affiliations:** 1 Department of Business Studies, Uppsala University, Box 513. SE-751 20, Uppsala, Sweden; 2 House of Governance and Public Policy, Stockholm School of Economics, Stockholm, Sweden; 3 Department of Global Public Health, Karolinska Institute, Stockholm, Sweden

**Keywords:** Global Health Diplomacy, Communities of Practice, Capacity Building, Situated Learning, Leadership

## Abstract

Addressing global health challenges requires complex coordination and collaboration between actors, often through the process of Global Health Diplomacy (GHD). Although considerable scholarship argues the importance of improving this process to build better health policies and systems, few studies have investigated the ‘health diplomats’ directly leading this work. In this study, we seek to better understand GHD from a practitioners’ view by exploring perceptions of knowledge acquisition, capacity building, and network development amongst those who coordinate and orchestrate global policy solutions. Taking an inductive qualitative approach, we conduct interviews of 54 experienced GHD professionals working across government, civil society, and private industry in 23 countries and identify key themes that outline challenges and opportunities for capacity building in GHD. Findings indicate a nascent global community bound by shared identity and motivations, but also hurdles regarding the transfer of tacit knowledge, network integration, and the improvement of institutional leadership. These findings highlight the boundaries by which knowledge and capacity are difficult for health diplomats to acquire or transfer, which help explain limitations to achieving better outcomes for global health. Further, this study may assist scholars and practitioners’ work by considering GHD as a purposeful community of practice.

## Introduction

Grand challenges—such as global health—require coordinated and integrated responses that draw on individual and institutional competencies, which may otherwise remain localized. Diplomacy can contribute to such coordination and integration, of which Global Health Diplomacy (GHD) addresses issues including public health goals.

Health diplomacy has its roots in the nineteenth century as a response to cross-border health risks [14]. When national policies in Europe failed to prevent the spread of disease, merchants grew frustrated their business activities were so affected by quarantines, they urged their governments to collaborate in international action (ibid). While health diplomacy still operates in a responsive manner to, for example, pandemic outbreaks, it has also been employed to preempt and even mitigate complex situations where health serves as a pretext for peace-keeping and soft-power [7, 26].

Both practitioners and scholars have increasingly recognized the importance of health in relation to other political issues such as trade, security, equity, development, and human rights [27]. This has led to recognition in the field of practice that health can also serve as an instrument of foreign policy [20, 50]. Many nations have established special offices responsible for health in their Foreign Services, employing attachés or even dedicated teams who coordinate between government offices and conduct diplomacy in an array of fora with foreign counterparties. GHD also extends beyond governments to include participation by civil society and private industry [26], and even a multisectoral approach at different levels within and outside the health sector [1].

However, GHD faces a general challenge due geopolitical tensions and a rise in nationalism, which have fuelled competitiveness rather than cooperativeness [27]. Even if GHD can develop a foundation for diplomatic relations in other political sectors, globalisation faces increasing criticism, and nations are coping differently with international relations [32]. This includes consequences for health and related policies such that “global collective action may be exacerbated in times of crisis given countries’ reflex of turning inwards […]” ([60], p. 218). The capacities and skills of health diplomats have direct consequence on the positioning of health in the global agenda, (and indirectly influence diplomacy in other areas of governance [31]. In the present multilateral setting, ‘diplomacy’ has presumably become more complex, such that “ensuring that diplomats have a combination of different skills increases the professionalism of global health diplomacy negotiations and the likelihood of achieving successful outcomes” ([27], p. 154).

This study aims to contribute to GHD as an interdisciplinary field of research by exploring practitioners’ experiences of knowledge acquisition, capacity building, and developing network to work effectively as ‘health diplomats To address these topics, we interviewed 54 experienced GHD practitioners, those who coordinate and orchestrate global policy solutions across this multilateral space. Given the multi-sectorial nature of this field, we also probe the nature and characteristics of a *community of practice* (CoP) amongst GHD practitioners, and, based on our findings, we theorize on further capacity building and the development of next-generation leadership for stronger GHD.

## Background

GHD engages different actors in international policy related to a wide range of health concerns. This relies upon “policy-shaping processes through which States, intergovernmental organizations, and non-State actors negotiate responses to health challenges or utilize health concepts or mechanisms in policy-shaping and negotiation strategies” ([36], p. 7). More broadly, GHD has been described as “the practices by which governments and non-state actors attempt to coordinate and orchestrate global policy solutions to improve global health” ([50], p. 61), spurred by collective realizations that with an interconnected world, global health challenges cannot be resolved by one country or agency alone.^1^ The interconnectivity between nations and their respective policies results in spill-over effects, such that “global health matters to domestic (national) health, and the foreign policies adopted by nations or negotiated internationally can have dramatic impacts on global health” ([52], p. 1080).

While the field has enlarged in size and scope, it has also become highly complex. The Millennium Development Goals adopted in 2000 helped position health at the center of multilateral discussions, bringing more actors into GHD [26]. This has added complexity to inter-organizational systems and collaborations by expanding the number of venues where GHD transpires, and to the process of coordinating and orchestrating solutions [18]. Further, there is no single, established definition for GHD, and prior investigations note that GHD lacks scholarly consensus and a theoretical foundation [50, 51]. Rather, GHD functions in multiple ways, namely as a *core* process between nations, as *multistakeholder* negotiations that may not lead to binding agreements, and *informal* engagements in the field between a wide array of actors [22]. Political changes have raised concern of the potential fragmentation in the field of global health, such that “rather than coordination we see a great deal of messiness” ([8], p. 245). This messiness, coupled with increased complexity, hamper the potential to address pressing global challenges [6, 60].

There is a wide array of social, cultural, political, and economic contexts consequent on health, and vice versa [19]. Hence, the individuals who navigate the diverse terrain of GHD require competencies and capacities encompassing these contexts to work together productively [3]. While GHD has been regarded as a fragmented, multi-sectorial field of research and practice in rapid change, there is an expectation amongst practitioners needing to understand and have capacity in this “problematic” space to enact positive impact [13].

Limited research has investigated these individuals involved in GHD processes [50] and extant studies show that a marriage of skills, competencies and networks are necessary for effective leadership. For example, ‘celebrity diplomats’ have employed their fame to help raise awareness of health-related issues [41]. National leaders play the pivotal function to achieve global initiatives, which relies upon committing their staff to coordinate and collaborate between Ministries of Health and Foreign Affairs [39] and institutional leaders serve as hubs of influence and information exchange across an international policy making community [60, 61]. By exploring more closely how and why health has become integrated into foreign policy, Gagnon and Labonté [31] stress the role of “tenacious policy entrepreneurs”, the individuals who link problems, policies, and politics streams and convene decision makers, initiate discussions, and influence the policy process.

Actors involved in health diplomacy thus rely upon such individuals for their knowledge and capacities. This knowledge and skill are particularly critical for effective leadership in complex GHD networks and the development of this field, as individuals able to gain attention and resources can better identify leverage points to foster the adoption of shared international norms [60, 61] as well as lobbying and framing [51]. However, it has been noted that some professionals joining GHD lack fundamental knowledge of the field, how to function within it, nor are they aware how to gain information or training [21], and why some states are absent from key international negotiations [33] While an array of capacity building programs for GHD exist [25, 44, 55], few studies have investigated their impact to the field.

### Communities of practice

Given the multi-sectorial nature of GHD and the important roles individuals play, it is critical to understand the field from a practitioners’ view. Such practitioners operate collectively to learn about institutions and processes with the purpose of building support for policy change by, for example, making productive use of networks [43] and forming teams of ‘insiders’ and ‘outsiders’ [42]. To investigate these collective activities, we probe the nature and characteristics of a *Community of Practice (CoP)* in GHD, defined as “a group of people who share a concern, set of problems, or a passion about a topic, and who deepen their knowledge and expertise in this area by interacting on an ongoing basis” ([59], p. 4).

The concept of CoP emerged within situated learning theory where individuals collaborate with peers to achieve a common purpose while gaining mastery through legitimate peripheral participation, i.e. the process newcomers learning from old-timers [35]. CoPs have since attracted much attention from scholars and practitioners interested in the role of situated practice in the process of learning and knowledge generation in organizations and professions, including health [2].

Scholarly work has sought to differentiate CoPs from other network forms in being characterized as self-organizing, informal, and self-selecting of both members and leadership [58], including shared passions and the deepening of knowledge and expertise through ongoing interaction [59]. Although Wenger redefined the concept over time, CoPs maintained consistent characteristics including “support for formal and informal interaction between novices and experts, the emphasis on learning and sharing knowledge, and the investment to foster the sense of belonging among members” [37]. Three generic structural characteristics of a CoP pertains to: (1) its *knowledge domain*: creating common ground, inspiring participation, and guiding participant learning, (2) its *community:* creating the social fabric that fosters interactions and willingness to share ideas, and (3) its *practice*: the specific focus around which the community develops, shares, and maintains its core of knowledge [59]. In a CoP, “members build their community through mutual engagement. They interact with one another, establishing norms and relationships of mutuality that reflect these interactions. To be competent is to be able to engage with the community and be trusted as a partner in these interactions” ([57], p. 4). Members of a CoP also need to “sustain dense relations of mutual engagement organized around what they are there to do” ([56], p. 74). CoPs can exist in diverse physical settings and can form and function virtually [9]. CoPs in health fields have been shown to generate and share knowledge, and to improve performance, including internationally [46].

While it is important to understand how knowledge capturing, sharing, and storing for capacity building operates across health-related fields [30], the global agenda necessitates understanding how capacity building between practitioners in health and in policy contribute to stronger health systems [29]. As these domains are bound in a process of diplomacy, it is notable that studies of CoPs in diplomacy show how these groups make significant political impact, not least by anchoring, refining, and innovating their practice in the face of challenges and uncertainty [4, 5]. However, while the importance of effective leadership in GHD is evidenced through a few studies [39, 60, 61], little is known about the development and regeneration of this leadership, and how practitioners find common purpose in their work, learn from one another, and develop innovative solutions in and around health policy.

As GHD practitioners operate between global health and policymaking, it is important to understand the role these practitioners occupy, their perceived challenges working together in the field, and mechanisms for future capacity building by asking: *To what extent does a community of practice exist within Global Health Diplomacy, and how is it manifested?* and the supporting queries:

- What are effective methods for capacity building for leadership in the Global Health Diplomacy community?
- What challenges in the field should future capacity building address?

## Methodology

### Research design

With the aim to answer these research questions through an ‘inside view’ of GHD, we sought to collect comprehensive, yet in-depth statements from practitioners in this field. Since perceptions may differ across individuals with various training and in different organizations, sectors and nations, a broad and diverse group of respondents was necessary. At the same time, in-depth data based on personal and professional experiences is needed when probing respondents’ subjective experiences and nuances of opinions regarding the research questions at hand. We therefore focused on collecting a broad set of qualitative data. Given the open-ended research questions with little or no preconceived categories or theories to base the study on, such an inductive research design was deemed preferable.

### Data

We conducted a series of semi-structured interviews to explore GHD participants' perceptions on various topics in a holistic and nuanced manner [28]. Respondents were identified using a first sample derived by two experts in the field with combined 60 years+ experience in GHD across 10+ countries, followed by snowballing method to expand the number of informants [49]. Sixty potential respondents were identified and contacted individually by an email explaining: the context for the study, who recommended their participation and why, and a request for their participation with a clear statement of opting in or out. Fifty-seven agreed to participate, yet 3 were eventually unavailable, leaving 54 participants who were interviewed during 2023.

An interview guide was crafted from a review of literature, and it functioned as a structured yet flexible framework. Prior to each interview, respondents confirmed their consent to recording, ensuring ethical considerations and following the Swedish legislation for ethical review (SFS 230:460) [34]. The same project members conducted all interviews where they made an introduction and overview of what respondents could expect from the session, reiterated the study topic, and emphasized respondents’ freedom to express themselves openly, using the questions as a guide to stimulate discussion. Each interview lasted 45-60 minutes and all were conducted in English over Zoom to facilitate audio and video recording, and then transcribed verbatim, yielding 685 A4 pages of text.

Understanding the characteristics and demographics of the respondents is essential for contextualizing study findings and drawing meaningful conclusions. We sought a purposive sampling approach to capture a range of perspectives, experiences, and backgrounds related to the research topic. While there is no formal, agreed definition of a “global health diplomat”, such persons have “a deep understanding of the ground realities and inequities that are inherent in global health…(making them) credible and authentic, and allows (them) to act as a bridge-builder and an ally, and to work to change the system from within” ([12], p. 1576). An additional selection criterium was a minimum of 5 years of experience in GHD as recognized by peers. We also sought diversity, with an aim for an equal distribution between genders (Male 56%, Female 44%) and drawing from 23 countries that span ‘Global North’ (56%) and ‘Global South’ (44%) [54]. Finally, we sought representation of the key actors in GHD - *Government, Private Industry* and *Civil Society* [26]* –* as well as *Academia* given that many in this community also actively influence and participate in GHD [18]. A summary of participants’ demographics in Table 1.

**Table 1:** Respondent demographics

***# Respondents***	54
***Professional Areas***	Academia: 14
	Government: 18
	Civil society: 17
	Private Industry: 5
***Gender***	Female: 24
	Male: 30
***Age Range***	32–75
***Geography***	Global South: 24
	Global North: 30

### Data analysis

Given the open-ended research question, we rely on an inductive coding process allowing for the emergence of common themes and distinct patterns in the data. Thematic analysis is the search for themes that emerge as being important to the description of a phenomenon [11]. This is a process of pattern recognition in the data, with identification of themes by “careful reading and re-reading of the data” ([48], p. 258) and where the emerging themes become the categories for analysis.

In the analysis we sought for interpretive rigor by explicating how interpretations of the data were achieved, and illustrating findings with quotations from the raw data ([48], p. 258) where respondents' reflections - conveyed in their own words – strengthen the validity and credibility of the research [45]. The process involved a systematic and iterative team approach of reviewing and coding (categorizing) the transcribed interviews. This allowed for the identification of key themes and subthemes. The first step involved a comprehensive review of all transcripts. Relevant quotes were identified and concept-coded based on the emergent patterns observed in the data. Iterative discussions in the research team led to the convergence of a coding manual, serving as a data management tool for organizing segments of similar or related text to assist interpretation [10]. Following the coding, quotes were clustered into themes, which facilitated a more focused analysis of each theme and a structuring and clean-up of the data. Each thematic area then underwent further analysis through additional review and discussion where themes were deconstructed into subthemes, allowing for a more nuanced understanding of the data. This iterative process ensured that no information was overlooked. Finally, the identified main points from each thematic area were synthesized into a coherent narrative by weaving together the various threads of analysis, merging, dividing, and drawing connections between the themes. Distinctions were made between verifiable descriptions of the field (key characteristics of GHD) our overall analysis of community members’ key perceptions (second-order themes) and finally, our thematic analysis of the community’s view on capacity-building needs (aggregate dimensions). All findings were discussed at length in the research team, facilitating the identification of meaningful insights and a broader understanding of the research phenomenon.

## Findings

**Fig. 1:**
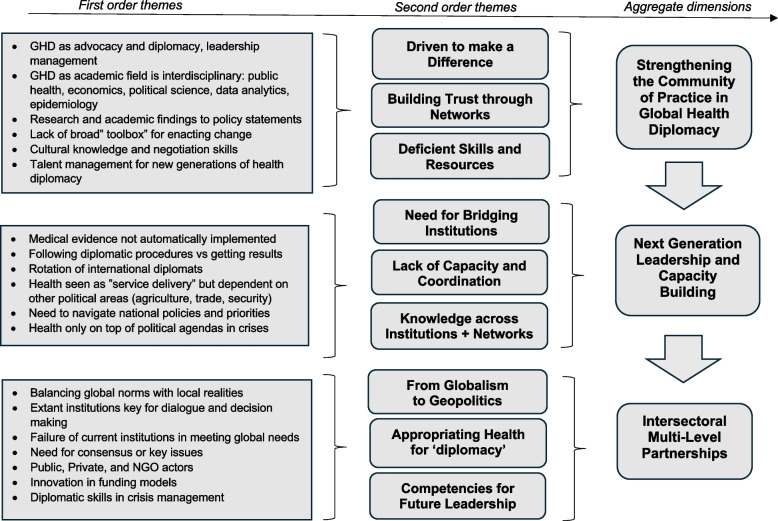
Capacity building in the community of practice of global health diplomacy

Our findings are portrayed in an analytical mapping (see Figure 1). In summary, individuals in GHD represent diverse educational and professional backgrounds but share strong convictions to improve policy development and implementation. While holding heterogeneous experiences, they describe themselves as self-driven to address multifaceted problems that affect whole populations and see themselves operating amongst fellow ‘health diplomats’ in that pursuit. They also seek new ways to strengthen their impact on global health, though feel challenged by resource scarcity, skill deficiencies, the complexity of institutions, and geopolitical tensions. Fostering future leadership is stressed as a vital capacity building mechanism on behalf of the community, with a variety of opinions of fostering such leadership.

### Driven to make a difference

Professionals’ entry into GHD is often unanticipated: “I never figured I would end up or thought I would even go into health diplomacy at all. But through the various positions that I held, it became of interest”. A large number of respondents trained as medical doctors, and many practiced medicine before holding leadership roles in organizations, universities, or governments. Even after many years away from medical practice, they continue to self-identify as a ‘surgeon’ or ‘physician’ in addition to other responsibilities: “I’m a medical doctor. I am also an academic in global health, and since 3.5 years now I am working in the Foreign Ministry.” Others were initially educated in areas including law, public policy, or public health. Such diversity amongst peers is acceptable and non-hierarchical in the community: “each of us has, perhaps, a different background.” While some had completed short trainings organized by their government or at an educational institute, most reported that they were “never trained in diplomacy” nor many of their current responsibilities, but rather they “learned through experience”.

Working in GHD involves ‘*double hatting*’ between multiple functions. As our respondents hold senior positions, they carry formal responsibilities ranging from public engagements in advocacy, negotiating, and influencing, to organizational duties like managing staff, reporting, and relationships with stakeholders like boards, elected officials, bureaucrats, partners, and funders. A common characteristic between them was achieving tangible outcomes, as one stated: *“I want to see results, numbers, real impact. I want to hear people saying ‘wow, life is so much better now’.”* Motivations to hold positions of responsibility and to tackle complex health problems are often driven by subjective experiences, including “*coming from an entrepreneurial family,*” studying in international schools, and witnessing intense health crises first-hand. Stepping into the ‘*fast changing and challenging roles’* of GHD requires ‘*courage’* and ‘*self*-*drive’* as such situations are often unanticipated and novel: “*I was younger than all the people that reported to me. I was the first woman to be director, and I was a social scientist. And suddenly in charge of a bunch of medics.*”

### Building networks for trust

Those working in GHD are expected to be familiar with key institutions in the field and be agile to engage with them. However, not all actors (including governments) have the resources to ensure their staff are trained in and comfortable understanding these structures: *“…it’s definitely challenging because institutions are incredibly complex and they change fast too, they are not passive*.” Instead, practitioners typically build up personal networks to learn “*how things really work and who to call*.”

While respondents align around a common motive that “*driving and improving access to health amongst underserved patient groups is the common denominator*,” the process to learn about GHD is not straightforward. Academic institutions have “*far more good applicants to our programs than we have space to accept*” and yet “*many from developing countries cannot get visas to join us*.” Many governments organize specialized training for junior staff to prepare for key international meetings, and some open this training to participants outside their governments: “*these sessions can be overwhelming. Lots of abbreviations. Lots of issues. But when they go to the real platform, they think ‘oh, this is the thing that I've heard before or something that lecturers informed us about in advance’…. The best benefit is to let them see the real platform, and the how each agenda links and how the meetings are conducted, what are the politics in the room, what are the relationships between countries or between the networks.”*

With regards to international fora like UN General Assemblies, respondents note that “the voice that gets to the room is the voice of the countries that have actually invested, having attachés in Geneva and New York”. Respondents agree that international fora are critical meeting points such that “the UN is the only framework we have” but also that an individual’s “patience, resilience and presence is what determines global diplomacy.” Turning to the regional and national levels, respondents stress the need to work both across governmental institutions and with external actors: “You want to go beyond the Ministry of Health or the health sector. You need to engage the highest level of government and the other sectors, and for this, diplomacy is critical.”

### Bridges to create impact

Respondents note that the science of health is merely a starting point, a necessary but insufficient condition to secure positive global health outcomes: *“evidence is not enough when engaging with politicians.*” In the realpolitik of international politics, academic knowledge only reaches so far: “*bridging between academia and the world of practice is usually a very thin and fragile and broken bridge.”* They stress that key task of GHD is to translate public health evidence towards actionable policies by communicating horizontally and laterally in a government apparatus: *“We [the community] need to foster the skills of negotiations with partners. Following up with partners, convincing partners dealing with politics of individual governments and regions. Speak languages that politicians can understand.”*

Advocacy, a core part of GHD, is also cumbersome. It involves working with global institutions and NGOs, but it requires local, regional, and national politicians who “*tend to operate in terms of election cycles*.” Misaligned agendas between different branches of the same government can lead to competition between individuals and groups for limited resources: “*in global health it's important to see us as a team and, to some extent, in the past we've been fighting against each other.*”

When advocating for global health initiatives, negotiating agreements, and engaging with diverse stakeholders, adapting communication is as a critical asset: *“You want your message to be simple. You want to repeat it. You want to choose certain words very carefully”*. This means garnering support with emotive messaging and storytelling: *“The facts will speak to the mind, but the stories will speak to the heart. In this age of very emotional issues like climate change, like pandemics, like conflicts...we need to be able to touch people's hearts in order to reach decisions and solutions.”*

A key practice is the ability to resonate with different audiences, but getting informed about these problems and audiences requires time to understand and work through. Ultimately, this is reinforced by the recognition of other perspectives and positions: “*that's where the power of this field is, that people come and sit around a table with all those different expertise. It also means filling in those gaps. It's not just any infectious disease physician off the street who can suddenly negotiate international agreements, right? And it's not like it's not any international negotiator who can suddenly figure out the ins and outs of the Nagoya protocol.*”

### Deficient skills and resources

A key theme in our interview data is that asymmetries of information at the individual and national level limit the building of stronger global cooperation. Respondents find their counterparts at health assemblies are often “*ill prepared*” or “*uniformed*” to dialogue about health issues. A “*lack of understanding*” of health results in its “*low priority*” in the agendas of key international fora, making the process of “*actually getting an initiative over the line so that you can implement it just impossible*”. While some nations can afford to send multiple delegates to these front-line discussions, many cannot. Investments to health are perceived as not generating the “*instant results*” associated with finance or trade, hence there is a lack of “*political commitment at the highest level of government.*” Consequently, many attachés marginalize health as a complex and unrewarding topic, or dismiss it as too difficult to learn about or address. This also stems from the frequent rotation of health officers in many institutions or governments, where staff “*are being exchanged after three or four years, and it takes three or four years until you even know your way around”*, making both interventions and reform challenging. Beyond this lack of experience with politically governed, multilateral, multi-sectorial cooperation as reason for lack of change, management practices are seen as pivotal to getting a job done well: *“What we don't have is the skill set. That famous toolbox.”*

### Lack of capacity and coordination

Despite the convictions and efforts of practitioners to develop and unify GHD, respondents mention structural limitations to what they can achieve. These include “*legal barriers*”, “*financial limitations*”, and tensions with “*local culture and values*” when attempting to address global challenges in a “*complicated world*” full of “*polarization*” and “*bickering across borders*”. A lack of unity at a supranational level leaves GHD respondents “*running in circles, because nobody knows who has to lead the governance of global health*”. Another primary challenge to GHD is a dependence of participation from elected politicians and funders, both of which have “*changing priorities*” that can compromise the development and sustainability of programs. Different actors “*do not speak the same language”* such that “*data sounds like common sense to a scientist. It doesn't for a politician*.” Respondents also report frustration with fundraising as “*donors are fickle*” in their commitment to an initiative, such that “*you start the program, and you don't have continuity because the funds are cut.*” Communication is another multifaceted issue, particularly at international fora or in building sessions as “*there are not enough resources for translations and translators*” which limits some to participate at all.

The most divisive issue amongst respondents is the participation of private industry. Many respondents working in civil society voiced concern about the rising influence of private firms and their motivations: “*companies come and say ‘Look, this is what we're doing, here are our programs and we hope it's interesting to you,’ but they haven't been really contributing to a debate. This is not very constructive; it is very much defending who they are. And I would be a little worried, you have to be cautious.”* Conversely, respondents in private industry felt blamed for global challenges and excluded from conversations, as one noted: “*I'm a cardiologist. I'm a professor. I also brought drugs to market that treat millions of patients. Who are they to moralize what I do? But is Big Pharma doing everything we can? The model can be different, both sides are wrong and that's what we need to solve.*”

### Knowledge across institutions

GHD is practiced through formal mechanisms and through informal relationship-based approaches, both of which are vital to master when learning how to “*play the GHD game”*. Attending fora where international negotiations, resolutions, and treaty development are conducted instruct how global health policies are confirmed, and informal exchanges contribute to the building of community: *“When you do bring people together, say on the margins of one of these other high-level meetings, it's relationship building more than anything else.”* Further, many educational institutions are influential sites where GHD practitioners gather for learning, reflection, and network building: “*the feedback we got about our program is that people want to meet each other, to take time out of their work-life to come and focus on this*.”

Maintaining personal networks and fostering collaborations beyond formal structures are vital to GHD as “*much of the work is addressed in private discussions*” like *“dinners in the evening.”* Despite the formality and protocols of fora, much of GHD is done through informal pathways between individuals, such as influencing decision makers in complex institutions by leveraging connections at all levels. For example: “*I never went to the ambassadors. I went to the lower-level staff...”* and “*to work with donors, you need to build credibility among those who do the hard work, who do the research, who collect the data, who write impressions for their bosses. I did the same thing with ministers.”*

Learning how to network and collaborate is achieved through informal mentorship where senior practitioners integrate progeny: “I try to create an enabling environment and give people their role and recognize their contribution…listen to them and give them a kind of sense of ownership”. While several respondents stress that “leaders must be visionaries,” prevailing practices do not necessarily support the development of creativity and visionary leadership: “The young ones come in often with more of an open mind, but then the system trains them in a certain way so that they become more formalized.”

### Gaps in the international arena

Current state-of-affairs in GHD is described “*a vacuum*” or “*a limbo*” due to lack of progress on the WHO Pandemic Agreement, and emerging geopolitical tensions. Diplomatic areas are being repositioned under the umbrella of national and regional security. Also, international organizations themselves face structural constraints to work together to address global health effectively: “*what we are getting is nothing progressive. We are just reaffirming old lines, and this is because of the same policy paralysis.*” Critiques like “*we have enough money to keep everyone healthy around the world. But the way we use it in structures that are duplicated and uncoordinated is a big problem*” and “*low-cost, effective interventions are available and can be made on a reasonable scale with reasonable impacts and in a short period of time. It's just that we don't think of that in much of health policy*” expose a lack of internal and inter-organizational coordination. Further, as “*each organization’s governance is very focused on the interests of the individual organization and not necessarily the interest of the system,*” incentives for change are out of scope.

Differences can also be vast between perspectives at the “*headquarters*” of organizations or governments and the “*people in the field*”. These differences result in misalignment of resources and create tensions, cited as key reasons for staff turnover and knowledge drain. Respondents lament their own weaknesses in leadership and management as “*we were never prepared*” and “*most of us are trained as clinicians*.” Despite their strong convictions, many feel that leadership roles in GHD require enormous resilience to face constant risk, ambiguity, and adversity such that: “*the person who has that skill can be a leader and the person who does not have that skill will be a participant*.”

### Appropriating health for ‘diplomacy’

All respondents voiced that health should be treated at a higher priority in global affairs, but perspectives differed in assigning responsibility, changing processes, or setting the sequence of priority. Some suggested more “*advocacy*” and “*proactivity*” amongst health diplomats to generate public awareness, others view “*institutional reform*” as a critical mechanism, while problems of “*coordination*” between governments, organizations and other stakeholders remain significant. It was also noted that public trust in global organizations and health authorities needs to improve, not least because “*there's no interest free space in global health*. *It is not a benign business. Health has its enemies*.”

There is also a need for improved communication between individuals: “we should assume that every player around the table has the same goal to improve people’s health. The pandemic demonstrated what you can achieve if you're willing to listen a little bit differently… we learned a lot how different players can collaborate with each other. They did that on a national scale and on an international scale. One should never underestimate what friendship does for listening to someone else's view.” Respondents believe this is why heath serves as an “icebreaking issue” between contentious parties to open or placate conversations around e.g. trade or security as the priority is mutually understood that “health among all peoples is fundamental to the attainment of peace and security.” As such, practitioners in GHD play an invaluable role to “enable the possibility of cooperation in highly volatile, complex environments.”

### Competencies for future leadership

Understanding how to “*navigate the formal rules and guidelines*” of organizational structures and political dynamics is stressed as "*fundamental to getting plugged into the system.”* We note that such capacity is typically achieved through “*experiential learning*” in the field, such as in-situ diplomatic experiences to put “*the political game”* on display. In addition, respondents call for more venues where emerging leaders can develop skills, self-awareness, and share experiences. As one said: *“We need established spaces for these future leaders to actually develop themselves, in particular a willingness to take risk and feeling ready to rock the boat.”* Another necessary, but not sufficient, condition for capacity building is the recruitment of people with specific motivations to participate in the GHD community - *“What we need is understanding, but also a certain passion.”* This passion can be nurtured, and it remains important to foster a sense of shared responsibility between actors: *“…the future of health diplomacy is all around. It’s not just the diplomat, but to do the diplomacy right, it's also scientists to do diplomacy with the diplomats who then negotiate the treaties. It's the civil society who must do diplomacy to hold governments accountable, etc.”*

New ways of working with “*digital technology and AI*” and a “*new generation of young people who think and act different*” excite current leaders to pass their knowledge along, and to see it employed in new ways. While all respondents articulated willingness to cooperate more, there are structural inequalities to how much can be achieved: “*There's a difference between having that lonely health attaché and the health attaché that has a team of three that has done significant research, gathered data, and has the full backing of their government to come in and influence and be part of the decision making*.”

## Discussion

By drawing upon the perceptions of experienced practitioners in this field, we have shown that ‘global health diplomats’ share a common domain of interest, interact regularly, and seek to improve their knowledge through these interactions. These interactions are both formal and informal in nature and venue, and these interactions have the purpose of forging mutual trust, building knowledge, facilitating collaboration with one another to achieve results.

These findings also indicate aspects of a community of practice consistent with Wenger [56, 57]. Further, members have self-selected into and defined aspects of leadership for this community [58] which has a knowledge domain established through a combination of passion-conviction and member guided capacity and network building [59].

The sharing of information and skills between persons across the loosely bound networks where they inform and enhance one another as ‘insiders’ is an important finding of our study [9, 42, 60, 61]. This includes not only nurturing ‘newcomers’ into mastery, it also keeps ‘old-timers’ relevant and performant in a dynamic system as it would facilitate efforts like framing and lobbying [51] which may rely upon tacit knowledge and the employment of language capable to persuade diverse stakeholders [24]. We also identify several limitations for opportunities to gain legitimate peripheral participation [35], including access to the fora (e.g. global assemblies) and decision makers (senior politicians) due to various structural inadequacies like finances, language skills, resource commitment, and coordination. Further, while institutional leaders and heads of state offer critical coordination and commitment for Global Health policy [39, 60, 61], it is reasonable to conclude that the implicated ‘diplomats’ must also hold adequate capacity to execute on their mandates.

Despite diverse national, educational, and professional backgrounds, we found practitioners unified in their values and goals around global health. GHD is highly collaborative for practitioners who are established in organizations and governments, yet the absence of formal training in management and leadership along the career development of ‘diplomats’ contributes to greater conditions of inefficiency, misalignment, and inflexibility. Simply requiring such training is also questionable: our findings contrast with Karačić Zanetti and Brown [21], who emphasize the need for clear career paths in GHD, which implies more formalization, which respondents criticized. Instead, GHD practitioners are characterized by their entrepreneurialism and leadership capacity, which are vital for positive institutional change [38, 60, 61]. This entrepreneurialism is, however, driven both by self-interest to contribute to goals shared across the community, but also as a problem-solving tool in reaction to a lack of formal training, opaque organizational structures and systems, and the limited number of people informed and available to participate in critical discussions of policy. These same reasons contribute to building network and credibility with “those who do the hard work” under key leaders and leveraging their support.

Leadership emerged as a central theme, with effective leaders characterized by their ability to frame complex issues, navigate political dynamics, and foster inclusive decision-making. 'Policy entrepreneurs' in global health possess knowledge and experience in both health and international relations, enabling them to connect within both contexts [17]. Our study supports this, highlighting a robust skill set and the agility of GHD practitioners. A sense of unity is evident among practitioners, driven by a shared commitment to problem-solving and policy implementation within a collaborative framework [58]. This collective ethos fosters an environment where practitioners are motivated by a desire to "make a difference" in global health outcomes. The shared identity among GHD practitioners is not only professional but often rooted in emotion and personal history. This extends the list of “specific attributes” required for GHD leadership in the policy process proposed by Gagnon and Labonté [31], and adds support to the consideration of a community of practice model where situated learning is exchanged with likeminded peers. Practitioners thus share a common domain of interest, interact regularly, and seek to improve their knowledge through these interactions [56, 57]. The profound commitment to transcend their job descriptions is so common that it is expressed as part of a defining identity, implying that community members both self-select and gain legitimacy by this intangible quality.

Respondents highlighted barriers to knowledge sharing, which hinder newcomers from gaining necessary capacities for leadership. These were blamed on lack of commitment by governments, including financial barriers, and logistical issues, namely visa restrictions. Addressing these barriers is essential for developing future leadership and preventing regionalism [55]. The spatial aspects of CoPs, where colocation facilitates group learning [15], underscore the importance of creating opportunities for face-to-face interactions despite these barriers [6]. Despites expanding digital tools for learning [44], these may lack the trust-building elements necessary for effective knowledge exchange [23]. Experiential learning and mentorship are paramount, emphasizing the importance of situated learning [35]. Respondents highlighted that knowledge and skills are earned through experiences at the sites where diplomacy occurs, while more academic trainings offer sites for network building and reflection [44]. Enhancing mastery through situated participatory activities, again supporting notions of a community of practice [35, 55].

Network relations facilitate professional growth, enabling practitioners to exchange knowledge, share best practices, and address complex health challenges collectively. This aligns with Wenger's [56, 57] view of CoPs as social structures where individuals with shared interests collaborate and learn from each other. Gabbay & May [16] also emphasize the advantage of utilizing formal and informal networks to frame evidence effectively, which suggests that CoPs offer a valuable learning space for observing and developing these skills. The widening of networks can also have positive influence on health policy as “broader community perspectives, identifying policy issues and generating potential solutions, and contributing unique insights” ([40], p. 9) which assist in knowledge translation, an area where our respondents noted competing priorities and diverse interpretations between stakeholders became problematic.

GHD is inherently interdisciplinary, involving stakeholders with diverse expertise. CoPs in GHD can drive innovation, promote synergy, and build resilience in health emergencies [20]. However, structural tensions with critical government leaders [39] who often "speak a different language" highlights the need for practitioners to be adept at translating or framing health issues into terms that resonate with political leaders [17]. While academic integration within the community is noted, practical application in a local context is central [25]. Bridging these gaps between academia and practice is crucial, particularly in fostering interdisciplinary collaborations [8, 60, 61].

Many respondents in government and civil society organizations were hesitant about the participation of private industry in GHD. Respondents’ concerns about potential conflicts of interest and the need for transparent and ethical engagement with private sector entities is ironic noting the fundamental role of private firms in the genesis of GHD [14], in global initiatives [26], and since the Covid-19 pandemic [19]. Similar concerns about the ‘oversized’ influence of private corporations have been echoed by scholars [32]. These may be an inevitable consequence in a multistakeholder governance of health, as participants coming from private sector bring the “toolkit” to negotiations lamented by many of our respondents. Similarly, differences of language and working culture may also be perceived as more distant between private corporations and other actors. However, previous investigations of health policy development processes offer caution based on evidence that private corporations exercise considerable influence on this process, but have ‘conflicts of interest’ that may compromise the public health outcomes sought by other stakeholders [1, 53].

Nurturing the next generation of GHD leaders through mentorship, experiential learning, and tailored leadership programs is vital. Leaders need technical expertise, interpersonal skills, emotional intelligence, and ethical conduct. Recognizing systemic barriers faced by marginalized groups is also important [6, 39]. Emphasis was placed on the value of hearing diverse perspectives and the necessity of inclusive practices. International collaboration is essential, as major shocks reveal weaknesses in global health systems and demonstrate the interdependencies of health, social, and economic structures [19]. GHD actors face numerous challenges, including resource constraints, competition, and political changes. While individual efforts drive GHD, collective action and higher coordination are necessary to achieve significant impact [37].

By bringing together more participants from across GHD actors, experiential learning can be enhanced, improving global health outcomes. The fostering a global community of practice where practitioners can share experiences, mentor each other, and build trust is paramount for an improved future of GHD.

## Limitations and conclusions

Notable literature on Communities of Practice in health-related fields [46] and diplomacy [5, 47] have helped generate and share knowledge from research, policy, and practice, yet there has been a dearth of such research of GHD. While this study has contributed insights to this growing field, we also acknowledge several limitations, which provide areas for future research. Our investigation explored perceptions from a strategic sampling of GHD, however, we were not able to gain access to respondents at multiple levels of the same organization, which may provide additional clarity about recruitment, mentorship, and boundary spanning activities. Further, while our study reveals the importance of networks and tacit knowledge transfer between participants, it would be valuable to further investigate how both co-located and virtual meeting-spaces function, and differ, as sites of learning and capacity building.

Finally, we selected respondents who were both self-acknowledged practitioners of GHD and validated as such by other respondents in our sampling process. Thus, the selection of respondents is relative to participation within a broad, but still specific, network of practitioners. Future research should explore, for example, practitioners at multiple levels of organizational structures (e.g. early career stage, mid-level managers) to understand team dynamics for capacity building, or geographic zones where health diplomacy is focused at a regional level to investigate barriers to global participation [6], and to better understand how access to information, digital platforms and virtual educational/training resources influence participation, network development and performance. As health diplomacy often takes place in “non-health focused, multilateral forums” [26] that include the private sector (e.g. multinational firms like pharmaceutical companies), more empirical studies of these actors’ engagement in GHD may add important insights to the growing literature in this field.

## Data Availability

No datasets were generated or analysed during the current study.

## Footnotes

1 Only one organisation – The World Health Organization (WHO) – has the explicit mandate to directing and co-ordinating international health work with the ability to adopt binding instruments [27].
